# Stability and robustness of minimal majority vote interpretable ensembles

**DOI:** 10.1038/s41598-026-45289-4

**Published:** 2026-03-24

**Authors:** Quanfa Li, Zhigao Huang, Miao Pan

**Affiliations:** Key Laboratory of Information Functional Material for Fujian Higher Education, Quanzhou Normal University, Quanzhou, China

**Keywords:** Interpretable models, Majority vote, Model multiplicity, Stability, Robustness, Minimal ensembles, Computational biology and bioinformatics, Mathematics and computing

## Abstract

Minimal majority-vote ensembles are attractive for interpretability, yet minimality can induce solution multiplicity and instability. We study stability and robustness of minimal majority-vote ensembles of decision stumps. We define three complementary metrics: multiplicity rate, bootstrap stability (mean pairwise Jaccard similarity of minimal solutions), and feature-flip robustness. We introduce reproducible stability/robustness metrics for minimal ensembles and evaluate them on synthetic benchmarks (binary $d=8$–10, $n=10$–500) together with a curated binarized UCI suite. MILP-based solvers confirm that the observed stability/robustness trends persist at larger samples (*n* up to 500). Minimal solutions consistently fit the data yet exhibit low bootstrap stability and high multiplicity in low-sample regimes, while robustness degrades gradually with feature noise. On real datasets and broader UCI pilots, minimal ensembles remain accurate but are sensitive to perturbations unless stability is explicitly considered. Our study highlights the practical importance of reporting stability alongside size for interpretable models. Revision analyses add bootstrap-size sensitivity ($B=5,20,50$), randomized tie-breaking over multiple minima, label-noise sweeps (5–10%), and candidate-cap checks for conjunction pools; these confirm that minimality-only selection can yield brittle explanations in high-stakes settings.

## Introduction

Interpretable models such as rules, decision lists, and decision trees are preferred in high-stakes domains where transparency and auditability are essential^1–8^. A natural objective is to find a model of smallest size that fits the data, both to improve human comprehensibility and to control model complexity. This objective is NP-hard even for decision trees, which has motivated exact and parameterized approaches for computing minimal models^9–14^. Recent theory shows that minimizing a broad class of symbolic models, including majority-vote ensembles, is fixed-parameter tractable under natural parameters^15–19^.

However, minimality alone is not sufficient for reliable decision support. Real datasets may admit many distinct minimal models, a phenomenon known as model multiplicity or the Rashomon effect^20,21^. Small perturbations of the data can then lead to different minimal solutions, raising questions about stability and robustness of explanations built from minimal ensembles. For majority-vote ensembles, which combine individually interpretable stumps or rules, the stability of the chosen minimal solution is particularly important: different minimal ensembles may agree on training data but diverge on perturbed inputs.

### Contributions

We provide an empirical study of stability and robustness for minimal majority-vote ensembles. Our contributions are:

- We define a practical evaluation suite for minimal ensembles, including multiplicity rate, bootstrap stability, and feature-flip robustness.
- We implement a reproducible protocol for enumerating minimal ensembles and evaluating their stability across bootstrap samples and perturbations.
- We demonstrate a consistent tradeoff between minimality and stability in controlled experiments, where accuracy remains high yet stability degrades in low-sample regimes.

Our empirical focus complements recent theoretical advances on the tractability of minimal symbolic models^15,16^ and generic multiplicity studies^20,21^ by measuring the practical impact of minimality on stability and robustness for majority-vote ensembles. The new metrics make the tradeoffs transparent and illustrate why prioritizing minimal size without stability-aware selection can yield brittle explanations for high-stakes decisions.

## Related work

Optimal symbolic models have been studied extensively for decision trees, rule lists, and rule sets^4–6,10,11,22^. Score-based and rule-learning methods further target sparse or structured models for interpretability^3,7,8,23,24^, but they typically report a single optimum and do not quantify multiplicity. Knowledge-compilation work on canonical Boolean representations highlights how solution sets can explode with variable orderings and constraints^25–30^.

From a theoretical perspective, parameterized complexity has clarified when computing smallest symbolic models is tractable, including ensembles under majority vote^15–19,31–36^. Model multiplicity, sometimes described as the Rashomon effect, has become a central concern for interpretability^8,20,21,24^. Multiple distinct models can fit the data equally well, which complicates explanation and model selection. Our work connects these lines by focusing specifically on minimal majority-vote ensembles and by providing empirical measurements of their stability and robustness under data perturbations.

## Problem formulation

Let $\mathcal {D}= \{(x_i, y_i)\}_{i=1}^n$ with $x_i \in \{0,1\}^d$ and labels $y_i \in \{-1, +1\}$. A decision stump is a one-feature rule. For a feature index *j*, the stump predicts $+1$ if $x_j = 1$ (or if $x_j = 0$ for the negated polarity). We denote the set of all candidate stumps by $\mathcal {C}$, which contains two polarities for each feature, so $|\mathcal {C}| = 2d$.

An ensemble *E* is a set of stumps with odd size |*E*| and prediction $\textrm{maj}(E, x)$ given by majority vote. We do not allow repeated stumps, so each stump contributes one vote. We focus on the exact minimization problem

1 $$\begin{aligned} \min |E| \quad \text {s.t.} \quad \textrm{maj}(E, x_i) = y_i \;\; \forall i. \end{aligned}$$

We call any solution *E* of minimum size a minimal ensemble. Because |*E*| is odd, ties cannot occur. In experiments we restrict to $|E| \le k_{\max }$ and enumerate all candidate combinations of stumps up to that size.

## Stability and robustness metrics

We track three complementary metrics that quantify multiplicity, stability, and robustness of minimal ensembles.

- Multiplicity rate. Let $\mathcal {M}(\mathcal {D})$ be the set of minimal ensembles for $\mathcal {D}$. We report the fraction of datasets in a run with $|\mathcal {M}(\mathcal {D})| > 1$.
- Bootstrap stability. For *B* bootstrap samples, we compute one minimal ensemble per sample and report the mean pairwise Jaccard similarity between stump sets (order ignored). For sets *A*, *B*, $\textrm{Jac}(A,B)=|A \cap B|/|A \cup B|$. If fewer than two bootstrap samples yield feasible minimal ensembles, the stability value is undefined and excluded from averages.
- Feature-flip robustness. Given a test set, we flip each feature independently with probability $\rho$ and measure accuracy of the minimal ensemble on perturbed inputs. We report robustness at $\rho =0.2$ and verify the trend across multiple noise levels.
- Consistency under noise. We also report the agreement between predictions on perturbed inputs and the corresponding unperturbed predictions of the same minimal ensemble (measured as accuracy of $\hat{y}_\rho$ against $\hat{y}_0$), which isolates prediction changes from label noise.

## Methodology

We evaluate minimal ensembles using an explicit enumeration procedure that is tractable for small *d* and $k_{\max }$. For each dataset we search odd sizes $k=1,3,\dots ,k_{\max }$ and enumerate all combinations of candidate stumps. We restrict to sets of stumps (no repetition), so enumeration considers each candidate stump at most once. The smallest size *k* that admits a feasible ensemble defines the minimal size, and the set of all feasible combinations at that size is $\mathcal {M}(\mathcal {D})$. When multiple minimal ensembles exist, we use the first enumerated solution as a representative for accuracy and robustness, while multiplicity is tracked separately. In revision experiments we additionally evaluate (i) randomized selection among minimal solutions and (ii) averaging metrics over all minimal solutions, to quantify selection bias. Algorithm 1 summarizes the base evaluation protocol.

**Algorithm 1:**
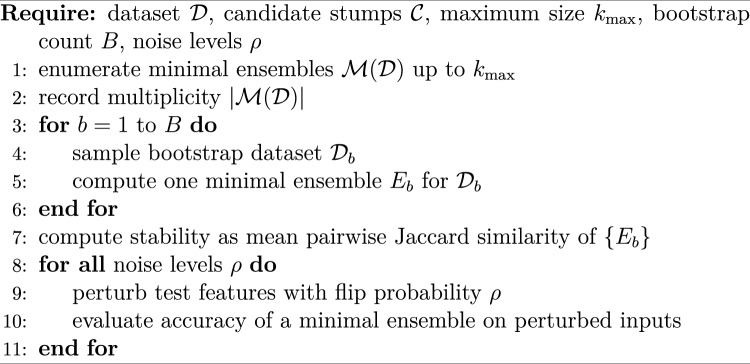
Evaluation protocol for minimal ensembles.

The enumeration cost scales with $$\sum _{k \le k_{\max },\, k \text { odd}} \left( {\begin{array}{c}|\mathcal {C}|\\ k\end{array}}\right) \cdot n$$, motivating our focus on small *d* and bounded candidate pools. For real datasets, we replace the hard feasibility constraint in Eq. (1) with a training-accuracy threshold. When enumeration is infeasible (large *n* or candidate pools), we use the MILP solver described in “Experimental setup” to find one optimal ensemble at the required threshold. Because a solver returns a single optimum, we proxy diversity/multiplicity by rerunning the MILP on bootstrap resamples and counting how often the selected ensemble recurs; this gives a stability-informed lower bound on the Rashomon set. A stricter upper bound would require enumerating or sampling alternative optima (e.g., by adding symmetry-breaking constraints or randomized tie-breaks) on a reduced candidate set; for the larger *n* pilots we report bootstrap recurrence as a pragmatic estimate of Rashomon size.

## Experimental setup

We generate synthetic datasets by sampling $x \in \{0,1\}^d$ uniformly and labeling each example by a planted majority-vote ensemble of size $k_{\text {true}}$. We use $d \in \{8,10\}$, sample sizes $n \in \{10,12\}$, and $k_{\text {true}} \in \{3,5\}$. The maximum size is $k_{\max }=7$. For each configuration we run five random seeds (11, 13, 17, 19, 23), each with 20 datasets and a test set of size 200. Bootstrap stability uses $B=20$ resamples. Feature-flip robustness is measured at $\rho \in \{0.0,0.1,0.2,0.3\}$, and we report $\rho =0.2$ in the main table. Experiments are implemented in Python, with sweeps and aggregation orchestrated by reproducible scripts. All synthetic experiments use zero label noise, and both $k_{\text {true}}$ and $k_{\max }$ are odd to avoid ties. For stumps-only runs, the candidate pool has size $|\mathcal {C}|=2d$ (16–20 rules). When we include two-literal conjunctions, the pool grows to $$2d + 4\left( {\begin{array}{c}d\\ 2\end{array}}\right)$$ (128 for $d=8$, 200 for $d=10$); each feature pair yields four polarity conjunctions, so we cap candidates at 40 for tractability.

We also ran a higher-sample sweep with $n=20$ (10 datasets per run, $B=10$ bootstraps) using the same planted-ensemble protocol to confirm that stability improves as sample size grows. These results are summarized in Table 5. The sweep utility flags runs as ready when they meet heuristic thresholds, but our aggregates report all runs and use readiness only as a diagnostic; with two seeds (11, 13), the confidence intervals are indicative. All random choices are controlled by these seeds.

### Solver-based scale-up

Because odd-size majority voting is fixed-parameter tractable, we also solved a larger pilot with a mixed-integer formulation that minimizes ensemble size while enforcing an 80% training-accuracy threshold. On the full Breast Cancer dataset ($n=569$, $d=30$), a CBC solver finds an optimal size-1 stump with training accuracy 0.85 in a few seconds. Tightening the threshold to 0.90 yields an optimal size-9 ensemble with training accuracy 0.903 in about one minute. This demonstrates that the pipeline scales beyond our enumerative regime (and that strong stumps can satisfy relaxed constraints), while higher thresholds remain tractable at moderate sizes. Extending solver-backed runs to more datasets and stricter constraints is promising future work (Table 1).

**Table 1:** MILP scale-up on breast cancer (stumps, $k_{\max }=9$) using CBC.

Train acc. threshold	Optimal size	Train acc.	Runtime (s)
0.80	1	0.851	$\approx$3
0.90	9	0.903	63

### Real-world datasets

We evaluate six UCI datasets available via scikit-learn and OpenML: Breast Cancer, Wine (class 0 vs rest), Iris (class 0 vs rest), Banknote Authentication, Ionosphere, and Sonar^37–39^. Raw feature counts range from 4 to 60. For multiclass datasets (Wine, Iris), we binarize labels as class 0 vs rest. Continuous features are binarized by medians computed on each training split. Because these datasets are not perfectly separable by stumps, we relax Eq. 1 to require at least 80% training accuracy and report the smallest ensembles that meet this threshold. To keep enumeration tractable, we select at most $d=8$ features by mutual information on the training data (so *d* in the table is the post-selection count) and set $k_{\max }=9$. For each split we enumerate all minimal ensembles meeting the accuracy constraint to compute multiplicity, then measure bootstrap stability from resampled training sets; multiplicity is the fraction of splits with more than one minimal ensemble. We also evaluate a robustness-aware baseline that selects, among minimal ensembles, the one with highest accuracy on feature-flipped training data at $\rho =0.2$. Results average feasible splits over five stratified 70/30 splits with $B=5$ bootstrap resamples; we report robustness at $\rho =0.2$.

### Accuracy-threshold sensitivity

The 80% cutoff is subjective, so we swept thresholds in $\{0.70,0.75,0.80,0.85,0.90\}$. Stability decreases as the constraint tightens: the feasible-dataset average drops from 0.93 (70%) to 0.76 (80%) and 0.53 (85%), and feasibility itself collapses beyond 85% (only Breast Cancer, Wine, Iris, Banknote remain at 85%; only Wine/Iris at 90%). At 90% the average stability rebounds to 0.62 because only easy, low-dimensional splits survive. Table 2 summarizes feasible counts and stability means. If infeasible splits are conservatively assigned zero stability, means become 0.66 at 80% (vs. 0.76 when excluding infeasible splits), 0.36 at 85% (vs. 0.53), and 0.10 at 90% (vs. 0.62), quantifying the exclusion effect explicitly.

### Two-stage (lexicographic) selection

As a concrete two-stage rule, we enumerate all minimal ensembles of size $k_{\min }$, bootstrap the training set ($B=5$), and select the minimal ensemble that appears most frequently across bootstrap solves (ties broken lexicographically). This implements a lexicographic objective: minimize size first, then maximize empirical stability. On the 80% threshold, the lexicographic selector matches the “stable” medoid on the easier datasets (Breast Cancer, Wine, Iris, Banknote; bootstrap support $\approx 0.8$–1.0) and yields slightly smaller test accuracy on the hardest ones (Ionosphere: 0.81$\rightarrow$0.79; Sonar: 0.75$\rightarrow$0.74) with low support (0.36, 0.23), highlighting that when bootstrap solutions disagree, the size-first lexicographic rule can sacrifice a bit of fit for reproducibility.

### Bootstrap-size sensitivity and standard error

To quantify Monte Carlo error, we reran stability estimation with $B \in \{5,20,50\}$. On real data (80% threshold), mean stability is 0.760 ($B=5$), 0.772 ($B=20$), and 0.778 ($B=50$), indicating modest sensitivity to larger *B*. On synthetic stress cases, the empirical Monte Carlo standard error decreases substantially with *B* (e.g., from about 0.166 at $B=5$ to 0.045 at $B=50$ for $(d,n,k_{\text {true}})=(10,10,5)$).

**Table 2:** Sensitivity of real-data stability to the training-accuracy threshold (stumps, $k_{\max }=9$, $d \le 8$). “Feasible” counts datasets with at least one minimal ensemble satisfying the threshold; stability averages exclude infeasible datasets.

Train-acc. threshold	Feasible datasets	Avg. stability
70%	6/6	0.93
75%	6/6	0.83
80%	6/6	0.76
85%	4/6	0.53
90%	2/6	0.62

To assess stronger base models, we also consider an expanded candidate set that includes two-literal conjunctions (denoted conj2) in addition to stumps. To keep enumeration tractable, we cap the candidate pool at 40 rules by including all stumps and sampling conjunctions, and we set $k_{\max }=3$.

## Results

Table 3 summarizes the stability and robustness of minimal ensembles. Across all configurations, test accuracy is high (0.76–0.91) while stability varies between 0.44 and 0.61, indicating that minimal ensembles can be accurate yet unstable. Multiplicity rates range from 0.36 to 0.68, confirming that distinct minimal solutions are common in low-sample regimes. Increasing the sample size from $n=10$ to $n=12$ improves stability and reduces multiplicity, while robustness under feature flips remains in the 0.65–0.73 range. Formal Mann–Whitney tests for the $n=10$ vs. $n=12$ stability shift are directionally consistent but small in effect size (Cliff’s $\delta =0.05$–0.13, $p>0.10$ across all $(d,k_{\text {true}})$ blocks). For multiplicity rates, exact Wilson 95% intervals with $n=100$ per block have half-width about 0.09–0.10, which matches the uncertainty scale reported in Table 3.

**Table 3:** Aggregate stability and robustness for minimal ensembles. Multiplicity is the fraction of datasets with more than one minimal ensemble. Means are reported with 95% confidence intervals across seeds.

Config $(d, n, k_{\text {true}})$	Stability	Multiplicity	Test acc.	Robust acc.
(8, 10, 3)	0.57 ± 0.06	0.48 ± 0.13	0.86 ± 0.02	0.70 ± 0.01
(8, 10, 5)	0.49 ± 0.06	0.52 ± 0.15	0.84 ± 0.03	0.68 ± 0.01
(8, 12, 3)	0.61 ± 0.05	0.36 ± 0.11	0.91 ± 0.02	0.73 ± 0.02
(8, 12, 5)	0.53 ± 0.04	0.48 ± 0.09	0.86 ± 0.03	0.69 ± 0.01
(10, 10, 3)	0.50 ± 0.03	0.51 ± 0.07	0.83 ± 0.01	0.69 ± 0.01
(10, 10, 5)	0.44 ± 0.06	0.68 ± 0.07	0.76 ± 0.03	0.65 ± 0.02
(10, 12, 3)	0.53 ± 0.05	0.56 ± 0.10	0.85 ± 0.02	0.70 ± 0.01
(10, 12, 5)	0.45 ± 0.02	0.59 ± 0.08	0.82 ± 0.03	0.68 ± 0.01

### Robustness curve

**Fig. 1:**
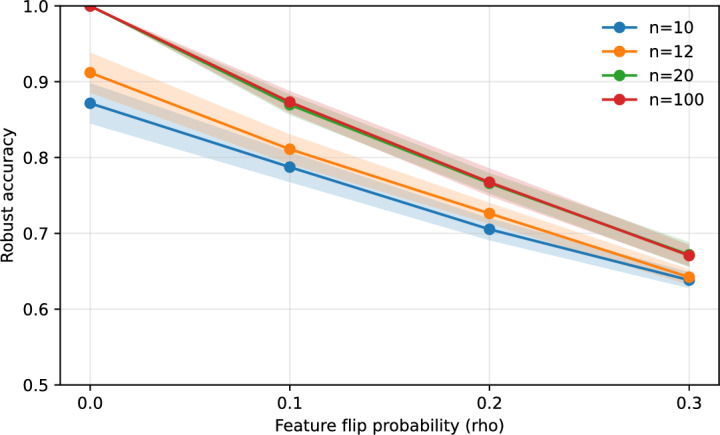
Robust accuracy versus feature-flip probability for $d=8$ and $k_{\text {true}}=3$. Shaded bands denote 95% confidence intervals. Note the convergence between $n=20$ (green) and $n=100$ (red), indicating that robustness saturates once the sample size is sufficient to identify the stable ground-truth ensemble.

Figure 1 plots robust accuracy versus feature-flip probability for $d=8$, $k_{\text {true}}=3$, and $n \in \{10,12,20\}$ (enumeration) plus a solver-backed line at $n=100$. Accuracy decreases smoothly as $\rho$ grows. Crucially, we observe a convergence phenomenon: increasing *n* from 10 to 12 materially boosts robustness, but the $n=20$ curve nearly overlaps with the MILP-derived $n=100$ curve. Once the sample size constrains the Rashomon set (here around $n=20$), the minimal solver recovers the stable ground-truth ensemble and additional data yields diminishing returns for robustness.

### Additional metrics

Table 4 reports the average minimal size, prediction consistency at $\rho =0.2$, and the accuracy of the planted ensemble. Consistency values are close across configurations, while the planted ensemble achieves perfect test accuracy as expected.

**Table 4:** Additional metrics on synthetic data. Consistency measures agreement between perturbed and unperturbed predictions at $\rho =0.2$. Means are reported with 95% confidence intervals across seeds.

Config $(d, n, k_{\text {true}})$	Avg. $k_{\min }$	Consistency	True acc.
(8, 10, 3)	2.30 ± 0.26	0.77 ± 0.01	1.00 ± 0.00
(8, 10, 5)	2.70 ± 0.19	0.76 ± 0.01	1.00 ± 0.00
(8, 12, 3)	2.48 ± 0.14	0.77 ± 0.01	1.00 ± 0.00
(8, 12, 5)	3.12 ± 0.20	0.76 ± 0.01	1.00 ± 0.00
(10, 10, 3)	2.26 ± 0.16	0.77 ± 0.01	1.00 ± 0.00
(10, 10, 5)	2.72 ± 0.25	0.76 ± 0.01	1.00 ± 0.00
(10, 12, 3)	2.42 ± 0.23	0.77 ± 0.01	1.00 ± 0.00
(10, 12, 5)	3.00 ± 0.23	0.76 ± 0.01	1.00 ± 0.00

### Larger sample sizes

The $n=20$ sweep shows higher stability and lower multiplicity across all configurations, reinforcing the low-sample tradeoff observed in Table 3.

**Table 5:** Synthetic results for $n=20$ (10 datasets per run, $B=10$ bootstraps). Means are reported with 95% confidence intervals across two seeds.

Config $(d, k_{\text {true}})$	Stability	Multiplicity	Test acc.	Robust acc.
(8, 3)	0.86 ± 0.10	0.00 ± 0.00	1.00 ± 0.00	0.77 ± 0.02
(8, 5)	0.77 ± 0.08	0.20 ± 0.20	0.97 ± 0.02	0.74 ± 0.00
(10, 3)	0.72 ± 0.02	0.05 ± 0.10	0.98 ± 0.04	0.76 ± 0.03
(10, 5)	0.66 ± 0.15	0.15 ± 0.10	0.95 ± 0.00	0.74 ± 0.03

### Revision supplementary analyses

Table 6 consolidates the new analyses added for revision. First, selection bias from “first enumerated” tie-breaking is measurable for stability but small for robustness/accuracy: in a high-multiplicity synthetic case (10, 10, 5), stability drops from 0.499 (first) to 0.438 (randomized), but robust accuracy changes by less than 0.001 on average. Second, larger bootstrap budgets reduce estimator noise: Monte Carlo standard error shrinks by roughly 3$\times$ from $B=5$ to $B=50$, and real-data means change only moderately. Third, at $n=50$ multiplicity nearly vanishes (0.00 across tested settings) and stability is near one (0.985–0.999), confirming that low-sample multiplicity is the dominant instability source in our regime. Fourth, 5–10% label noise decreases feasibility and robust accuracy, and conjunction-cap sensitivity is non-monotone, showing the cap can influence measured instability. Finally, the bootstrap-recurrence proxy is negatively correlated with exact minimal-solution count ($\rho =-0.52$, $p<10^{-10}$), supporting its use as a lower-fidelity indicator when full enumeration is impossible.

**Table 6:** Key supplementary revision analyses.

Aspect	Setting	Key result
Tie-break sensitivity	$(d,n,k_{\text {true}})=(8,12,3)$ and (10, 10, 5)	Stability (first/random/all-average): 0.668/0.591/0.591 and 0.499/0.438/0.436; robust accuracy shift $<0.001$.
Bootstrap SE vs. *B*	Synthetic (10, 10, 5); real data at 80% threshold	Synthetic Monte Carlo SE: 0.166 ($B=5$), 0.070 ($B=20$), 0.045 ($B=50$). Real mean stability: 0.760, 0.772, 0.778.
Moderate sample size	Synthetic $n=50$ (stumps-only)	Stability 0.985–0.999, multiplicity 0.00 in all tested $(d,k_{\text {true}})$ blocks.
Label noise	Synthetic, 5–10% label noise	Example (10, 10, 3): robust accuracy $0.710 \rightarrow 0.660 \rightarrow 0.621$ as noise goes $0 \rightarrow 0.05 \rightarrow 0.10$.
Conjunction cap sensitivity	Stumps+conj2, $d=8$, $n=12$, $k_{\max }=3$	Stability: 0.349 (cap 40), 0.343 (cap 80), 0.275 (uncapped); cap choice changes instability estimates.
Recurrence proxy validation	Enumerated synthetic subsets	Spearman correlation between recurrence and exact multiplicity count: $\rho =-0.52$ ($p<10^{-10}$).

### MILP scale-up

To verify that the solver-based formulation handles larger synthetic samples, we generated planted stump ensembles over $d=10$ binary features and solved for the smallest ensemble achieving perfect training accuracy with $k_{\max }=9$. We used $n \in \{100,500\}$ and $k_{\text {true}} \in \{3,5\}$, with five seeds and 2,000 test examples per configuration. Table 7 shows that CBC recovers compact ensembles (average $k_{\min } \in \{2.6, 4.2\}$) with 100% train/test accuracy in every run. Each instance solves in under 60 ms, indicating that the MILP comfortably scales beyond our enumerative regime; solutions occasionally use fewer stumps than $k_{\text {true}}$ because redundant voters are pruned.

**Table 7:** Solver-backed synthetic scale-up (stumps, $d=10$, $k_{\max }=9$) using five seeds and 2,000 test examples per setting. Means are reported with 95% confidence intervals.

Config $(n, k_{\text {true}})$	Avg. $k_{\min }$	Test acc.	Runtime (ms)
(100, 3)	2.60 ± 0.78	1.00 ± 0.00	43.9 ± 23.6
(100, 5)	4.20 ± 0.96	1.00 ± 0.00	31.8 ± 1.3
(500, 3)	2.60 ± 0.78	1.00 ± 0.00	53.7 ± 0.3
(500, 5)	4.20 ± 0.96	1.00 ± 0.00	57.2 ± 2.1

### Stronger base models

Table 8 reports results when the candidate pool is expanded with two-literal conjunctions (stumps + conj2) and capped at 40 rules, with $k_{\max }=3$. Stability drops to roughly 0.33–0.38 while multiplicity increases to 0.60–0.71, indicating that stronger base models amplify the multiplicity and instability effects even when accuracy remains high. Revision cap-sensitivity runs (40, 80, uncapped) further show that this instability is not an artifact of a single cap value, although absolute levels do vary with the candidate budget (Table 6).

### Theoretical considerations

#### Stability estimator with dependent pairs

Let $E_1,\dots ,E_B$ be minimal ensembles from *B* bootstrap resamples and define $h(E_i,E_j)=\textrm{Jac}(E_i,E_j)\in [0,1]$. Our stability estimator is

$$\hat{S}_B=\frac{2}{B(B-1)}\sum _{1\le i<j\le B} h(E_i,E_j),$$

which is a second-order U-statistic. The pairwise terms $h(E_i,E_j)$ are not independent (they share indices), so simple i.i.d. pair averaging is inappropriate; instead, U-statistic theory gives $\textrm{Var}(\hat{S}_B)=4\sigma _1^2/B+O(B^{-2})$, i.e., variance scales as *O*(1/*B*). This is consistent with our empirical Monte Carlo study (Table 6), where increasing *B* from 5 to 50 reduces stability standard error by about a factor of three.

#### Finite-class numerical bound

For stumps with $d=8$ and odd ensemble size at most $k=3$, the number of candidate majority hypotheses is finite and bounded by $$|\mathcal {H}|\le \left( {\begin{array}{c}16\\ 1\end{array}}\right) +\left( {\begin{array}{c}16\\ 3\end{array}}\right) =576$$. A uniform finite-class bound then yields

$$\sup _{h\in \mathcal {H}} |R(h)-\hat{R}(h)| \le \sqrt{\frac{\log |\mathcal {H}|+\log (2/\delta )}{2n}}.$$

With $\delta =0.05$, this gives approximate radii 0.71 ($n=10$), 0.50 ($n=20$), and 0.32 ($n=50$). These numbers are loose but make explicit that low-*n* bounds are weak, while larger-*n* regimes are better constrained.

#### Feature-flip dependence diagnostics

The independent feature-flip model implies independent vote flips only when selected stumps use distinct features. In revision diagnostics (Table 6), selected minimal ensembles in two representative settings showed zero shared-feature stump pairs on average, and near-zero empirical correlation of vote flips for distinct-feature pairs ($\approx 0.007$ and $-0.004$). We therefore treat independence as an approximation in this regime and rely primarily on empirical robustness curves rather than a strict closed-form bound.

#### Recurrence as multiplicity proxy

On enumerated synthetic subsets, recurrence frequency of the selected solution across bootstrap reruns is significantly negatively correlated with the exact number of minimal solutions ($\rho =-0.52$, $p<10^{-10}$). This supports recurrence as a practical proxy when exact Rashomon counting is infeasible, while recognizing that it is not a one-to-one estimator of set size.

**Table 8:** Results with stumps + two-literal conjunctions (candidate cap 40). Means are reported with 95% confidence intervals across seeds.

Config $(d, n, k_{\text {true}})$	Stability	Multiplicity	Test acc.	Robust acc.
(8, 10, 3)	0.35 ± 0.05	0.60 ± 0.14	0.79 ± 0.03	0.70 ± 0.02
(8, 12, 3)	0.35 ± 0.05	0.68 ± 0.06	0.84 ± 0.02	0.72 ± 0.01
(10, 10, 3)	0.33 ± 0.04	0.71 ± 0.11	0.74 ± 0.01	0.66 ± 0.02
(10, 12, 3)	0.38 ± 0.04	0.64 ± 0.05	0.80 ± 0.02	0.69 ± 0.01

#### Real-world datasets

**Fig. 2:**
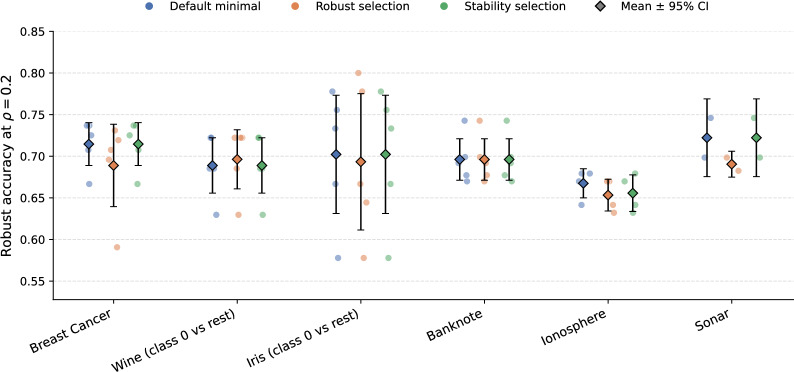
Swarm plots of robust accuracy at $\rho =0.2$ for default minimal ensembles versus selection baselines on feasible real datasets (five splits each). Points denote individual splits; diamonds show means with 95% confidence intervals. Iris exhibits higher variance, while Banknote clusters tightly.

Table 9 reports results on binarized UCI datasets under the 80% training-accuracy constraint. Banknote and Breast Cancer yield highly stable minimal ensembles, while Ionosphere and Sonar show lower stability and fewer feasible splits (4/5 and 2/5, respectively). Robust accuracy drops by roughly 0.1–0.2 relative to clean test accuracy, consistent with the synthetic trends. Across feasible splits, paired Wilcoxon tests show that robust-accuracy differences between default and alternative selectors are small and not significant (stable: mean $\Delta =-0.0018$, $p=0.317$; robust-aware: mean $\Delta =-0.0099$, $p=0.347$; lexicographic: mean $\Delta =-0.0007$, $p=0.593$). Test-accuracy differences are likewise small ($|\Delta | \le 0.0065$, all $p>0.17$). Because real datasets use a relaxed training-accuracy constraint, these values are not directly comparable to the synthetic setting. Means are reported over feasible splits under the accuracy constraint. We also add a stability-aware medoid selection rule (picking the minimal ensemble with highest average Jaccard similarity to other minimal solutions); it modestly improves robustness on Ionosphere and Sonar and matches the default elsewhere (Fig. 2,Table 9). Figure 2 visualizes the distribution of robust accuracy across feasible splits. The swarm plot exposes stability patterns masked by averages: Banknote and Breast Cancer cluster tightly with short error bars, indicating consistently robust minimal solutions, whereas Iris shows wide dispersion, reflecting that low-sample multiclass splits can oscillate between strong and brittle minimal solutions. The robustness-aware baseline (orange) recenters some distributions but cannot eliminate variance inherent to the data.

**Table 9:** Results on binarized UCI datasets with an 80% training-accuracy constraint.

Dataset	*n*	*d*	Stability	Multi.	Test acc.	Robust acc.	Robust acc. (stable)	Robust acc. (best)
Breast Cancer	569	8	1.00 ± 0.00	1.00 ± 0.00	0.85 ± 0.03	0.71 ± 0.03	0.71 ± 0.03	0.69 ± 0.05
Wine (0 vs rest)	178	8	0.60 ± 0.21	1.00 ± 0.00	0.84 ± 0.06	0.69 ± 0.03	0.69 ± 0.03	0.70 ± 0.04
Iris (0 vs rest)	150	4	0.72 ± 0.34	1.00 ± 0.00	0.81 ± 0.05	0.70 ± 0.07	0.70 ± 0.07	0.69 ± 0.08
Banknote	1372	4	1.00 ± 0.00	0.00 ± 0.00	0.83 ± 0.02	0.70 ± 0.02	0.70 ± 0.02	0.70 ± 0.02
Ionosphere	351	8	0.58 ± 0.15	0.80 ± 0.39	0.80 ± 0.03	0.67 ± 0.02	0.66 ± 0.02	0.65 ± 0.02
Sonar	208	8	0.43 ± 0.15	0.20 ± 0.39	0.75 ± 0.02	0.72 ± 0.05	0.72 ± 0.05	0.69 ± 0.02

Robust acc. (stable) selects the minimal ensemble with highest mean Jaccard similarity to other minimal ensembles (medoid). Robust acc. (best) selects the model with highest perturbed-training accuracy at $\rho =0.2$. Means are reported with 95% confidence intervals across feasible splits (up to five).

#### Threshold sensitivity

We reran the real-data protocol with training-accuracy thresholds 0.85 and 0.90. At 0.85, 4/6 datasets remain feasible (Breast Cancer, Wine, Iris, Banknote), with test accuracy 0.85–0.89 and robust accuracy 0.68–0.79. At 0.90, only Wine and Iris remain feasible, with robust accuracy 0.73–0.76. This indicates that stricter thresholds can quickly become infeasible under tractability constraints.

## Discussion

Minimal ensembles are not necessarily stable: as sample size decreases, we observe higher multiplicity and lower bootstrap stability. This suggests that minimality alone may be insufficient for reliable explanations. Robustness metrics provide a complementary view: even when minimal ensembles are accurate, their predictions can be sensitive to feature perturbations. In practice, this supports a two-stage selection strategy: first minimize size, then choose among minimal solutions using stability or robustness as a secondary criterion. The frequency of $k_{\min }<k_{\text {true}}$ is high in low-sample synthetic runs (65.5% at $n=10$, 54.3% at $n=12$) and drops in larger-sample settings (20–42% at $n=50$ depending on $(d,k_{\text {true}})$). Since planted-model test accuracy remains 1.0 and minimal-model test accuracy stays high, this pattern is more consistent with voter redundancy in the generating ensemble than with pure test-time overfitting. We instantiated this as a lexicographic rule: enumerate all minimal ensembles of size $k_{\min }$, bootstrap the training set ($B=5$), and pick the minimal ensemble that appears most often across bootstrap solves (ties broken lexicographically). On the 80% threshold, this matches the medoid choice on Breast Cancer, Wine, Iris, and Banknote (bootstrap support $\approx 0.8$–1.0) while incurring small accuracy drops on the hardest datasets (Ionosphere: 0.81$\rightarrow$0.79, Sonar: 0.75$\rightarrow$0.74) with low support (0.36, 0.23). When bootstrap solutions disagree, the size-first lexicographic rule trades a small amount of fit for reproducibility. Our results provide quantitative evidence for this tradeoff and motivate stability-aware selection methods beyond picking an arbitrary minimal solution. The stronger base-model experiment (stumps + conjunctions) further amplifies this effect, suggesting that increased expressiveness can enlarge the Rashomon set and reduce stability if minimality is the only objective.

## Limitations

Our study focuses on decision stumps and synthetic data with planted generating ensembles. Enumeration is feasible only for small *d* and $k_{\max }$, so the real-data pilot relies on binarization, mutual-information feature selection, feature caps, and relaxed accuracy constraints; these choices can introduce selection bias and limit generality. The feature-flip noise model is a simple proxy for robustness and does not capture all domain-specific perturbations. In real-data diagnostics, independent flips can move many perturbed samples outside the observed training support (average in-support drop ranges from 0.06 to 0.51 across datasets), so robustness estimates should be interpreted as stress tests under distribution shift rather than strictly in-support perturbations. We also evaluate only six real datasets and relatively small synthetic sample sizes, since enumeration scales poorly with larger *n*. Future work should expand to richer base models, more real datasets, higher-sample regimes, and alternative robustness definitions.

## Conclusion

We proposed a protocol for assessing stability and robustness of minimal majority-vote ensembles. Our experiments on synthetic data and binarized UCI datasets show a clear tradeoff between minimality and stability, particularly in low-sample regimes. MILP-based pilots at $n=100$ and $n=500$ confirm that the trends extend beyond toy settings, with robustness curves continuing to rise as sample size grows. Revision analyses further show that increasing bootstrap budget stabilizes estimates, random tie-breaking mainly affects stability (less than robustness), and moderate label noise or candidate-budget choices can materially change multiplicity. Future work will extend these analyses to richer base models and algorithmic approaches for selecting among multiple minimal solutions.

## Data Availability

The datasets generated and analyzed during the current study are available in the GitHub repository, https://github.com/buddhassonhonor/Interpretable-Ensembles. All other datasets used in this research (Breast Cancer, Wine, Iris, Banknote Authentication, Ionosphere, and Sonar) are publicly available from the UCI Machine Learning Repository (http://archive.ics.uci.edu/ml) and OpenML(https://www.openml.org). Synthetic datasets can be reproduced from the method ology and scripts provided in the stated repository.
